# Nanotechnology in the real world: Redeveloping the nanomaterial consumer products inventory

**DOI:** 10.3762/bjnano.6.181

**Published:** 2015-08-21

**Authors:** Marina E Vance, Todd Kuiken, Eric P Vejerano, Sean P McGinnis, Michael F Hochella, David Rejeski, Matthew S Hull

**Affiliations:** 1 Institute for Critical Technology and Applied Science, Virginia Tech, 410 Kelly Hall (0194), 235 Stanger St., Blacksburg, VA 24061, United States; 2 Woodrow Wilson International Center for Scholars, One Woodrow Wilson Plaza - 1300 Pennsylvania Ave., NW, Washington, DC 20004, United States; 3 Department of Civil & Environmental Engineering, Virginia Tech, 418 Durham Hall (0246), Blacksburg, VA 24061, United States; 4 Department of Materials Science and Engineering, Virginia Tech, Holden Hall (0237), Blacksburg, VA 24061, United States; 5 Department of Geosciences, Virginia Tech, 4044 Derring Hall (0420), Blacksburg, VA 24061, United States

**Keywords:** consumer products, database, inventory, nanoinformatics, nanomaterials

## Abstract

To document the marketing and distribution of nano-enabled products into the commercial marketplace, the Woodrow Wilson International Center for Scholars and the Project on Emerging Nanotechnologies created the Nanotechnology Consumer Products Inventory (CPI) in 2005. The objective of this present work is to redevelop the CPI by leading a research effort to increase the usefulness and reliability of this inventory. We created eight new descriptors for consumer products, including information pertaining to the nanomaterials contained in each product. The project was motivated by the recognition that a diverse group of stakeholders from academia, industry, and state/federal government had become highly dependent on the inventory as an important resource and bellweather of the pervasiveness of nanotechnology in society. We interviewed 68 nanotechnology experts to assess key information needs. Their answers guided inventory modifications by providing a clear conceptual framework best suited for user expectations. The revised inventory was released in October 2013. It currently lists 1814 consumer products from 622 companies in 32 countries. The Health and Fitness category contains the most products (762, or 42% of the total). Silver is the most frequently used nanomaterial (435 products, or 24%); however, 49% of the products (889) included in the CPI do not provide the composition of the nanomaterial used in them. About 29% of the CPI (528 products) contain nanomaterials suspended in a variety of liquid media and dermal contact is the most likely exposure scenario from their use. The majority (1288 products, or 71%) of the products do not present enough supporting information to corroborate the claim that nanomaterials are used. The modified CPI has enabled crowdsourcing capabilities, which allow users to suggest edits to any entry and permits researchers to upload new findings ranging from human and environmental exposure data to complete life cycle assessments. There are inherent limitations to this type of database, but these modifications to the inventory addressed the majority of criticisms raised in published literature and in surveys of nanotechnology stakeholders and experts. The development of standardized methods and metrics for nanomaterial characterization and labelling in consumer products can lead to greater understanding between the key stakeholders in nanotechnology, especially consumers, researchers, regulators, and industry.

## Introduction

Advancements in the fields of nanoscience and nanotechnology have resulted in myriad possibilities for consumer product applications, many of which have already migrated from laboratory benches into store shelves and e-commerce websites. Nanomaterials have been increasingly incorporated into consumer products, although research is still ongoing on their potential effects to the environment and human health. This research will continue long into the future.

To document the penetration of nanotechnology in the consumer marketplace, the Woodrow Wilson International Center for Scholars and the Project on Emerging Nanotechnology created the Nanotechnology Consumer Product Inventory (CPI) in 2005, listing 54 products [1]. This first-of-its-kind inventory has become one of the most frequently cited resources showcasing the widespread applications of nanotechnology in consumer products. In 2010, the CPI listed 1012 products from 409 companies in 24 countries. Even though it did not go through substantial updates in the period between 2010 and 2013, it continued being heavily cited in government reports [2] and the scientific literature – the website http://www.nanotechproject.org has been cited over 2,580 times in articles according to Google Scholar – and became a popular indicator of the prevalence of nanotechnology in everyday life and the need to further study its potential social, economical, and environmental impacts [3–6]. The CPI has also been criticized due to its lack of science-based data to support manufacturer claims. Other longstanding suggestions for improvement included: more frequent updates, indications when products were no longer available for purchase by consumers, and the inclusion of more product categories to improve the searchability of the CPI database [7].

Since the creation of the CPI, other nanotechnology-related inventories have been developed around the world. In 2006, a German company launched a freely accessible internet database of nanotechnology products [8]. The website associated with this database was not accessible at the time of this writing and its last available record is from May 2014, when 586 products were listed. In 2007, Japan’s National Institute of Advanced Industrial Science and Technology created an inventory of “nanotechnology-claimed consumer products” available in Japan [2]. This inventory is freely accessible online and it acknowledges the CPI in its website. At the time of this writing, the inventory listed 541 product lines and 1241 products; its last update occurred in 2010 [9]. In 2009, two European consumer organizations, the European Consumers Organization (BEUC) and the European Consumer Voice in Standardization (ANEC), joined efforts to develop an inventory of “consumer products with nano-claims” available to consumers in Europe [10]. A new inventory was generated annually from 2009 to 2012, but the 2011 and 2012 versions focused exclusively on products containing silver nanoparticles (nanosilver); the latest version in 2012 listed 141 nanosilver products. This inventory does not provide a searchable online database, but it can be downloaded for free as an Excel spreadsheet. In 2012, the Danish Consumer Council and Ecological Council and the Technical University of Denmark’s Department of Environmental Engineering launched “The Nanodatabase”, an inventory of products available for purchase that are claimed to contain nanomaterials and are available in the European consumer market [11]. This inventory has been continually updated and it currently lists 1423 products.

These worldwide efforts to understand the transition of nanotechnology from the laboratory bench to the commercial marketplace substantiate the need for applying the concept of nanoinformatics to a nanotechnology-enabled consumer products database, which is to determine the most relevant and useful information needed by a variety of stakeholders and to develop tools for its most effective use [12]. Databases such as the CPI offer information useful and relevant to a variety of stakeholders who are interested in a) understanding which consumer products incorporate nanotechnology and b) developing strategies, tools, and policies that may be needed to ensure safe and responsible use of those products.

Nanomaterials are regulated without specific provisions in the U.S. as hazardous chemical substances and pesticides, under the EPA’s Toxic Substances Control Act (TSCA) [13] and the Federal Insecticide, Fungicide, and Rodenticide Act (FIFRA) [14]. When used as food additives, drugs, or cosmetics, nanomaterials are regulated under the Federal Food, Drug, and Cosmetic Act (FFDCA).

In the European Union, nanomaterials are regulated under the Concerning the Registration, Evaluation, Authorization and Restriction of Chemicals (REACH) and the Classification, Labeling, and Packaging (CLP) regulations when those are classified by the Commission as hazardous chemical substances [15]. The Biocidal Products Regulation (BPR) has special provisions for biocidal materials that consist of nanoparticles, aggregates, or agglomerates in which at least 50% of primary particles have at least one dimension between 1 and 100 nm, with no provisions for “novel properties” stemming from their small size [16]. Cosmetics that contain nanomaterials are also regulated by the European Commission, and although the use of nanoscale titanium dioxide is permitted, zinc oxide is not [17]. The German Federal Environment Agency performed an Impact Assessment of a European Register of Products Containing Nanomaterials and determined that when compared to the implementation of a variety of national registries, an unified European registry would bring many advantages, including a lower cost for industries and, ultimately, a registry would benefit consumers, companies, and governments [18].

The objective of this work was to modify the CPI to improve its functionality, reliability, and utility to the diverse group of stakeholders who have come to depend on it as a critical resource for current information on nano-enabled consumer products. Specific objectives were (1) to update the CPI data to gain an insight into the penetration of nanotechnology in the consumer products market over the past decade; (2) to determine and implement improvements to the CPI based on the scientific literature and a survey of nanotechnology experts and CPI users; and to (3) develop a sustainable model to facilitate future CPI maintenance using crowdsourcing tools.

Below, we present a brief history of this inventory over a decade of existence. We also describe the specific changes made in the inventory during this project (referred here as CPI 2.0). Finally, we present an overview of the current data present in the CPI after the completion of this project.

## Results and Discussion

### CPI growth over time

Table 1 lists the growth of the CPI since 2005. In 2011, before this current project, the CPI described 1314 products. Since then, 489 products that are no longer available or marketed as containing nanotechnology have been archived and 500 products have been added. The new total of 1814 products as of March 2015 represents a thirty-fold increase over the 54 products originally listed in 2005 – which is not a complete representation of the growth of this market, as our methodology has also evolved over time. Based on our review, the CPI is the largest online inventory of nanotechnology consumer products available. Products come from 622 companies located in 32 countries (Supporting Information File 1, Table S1).

**Table 1:** Number of products in the CPI over time.

Year	Total products	Products added	Products archived	Data collection notes

2005	54	54	0	Beginning of CPI as a static pdf document.
2006	356	302	0	Launch of the online CPI.
2007	580	278	0	Nanoscale silver emerged as most cited nanomaterial.
2008	803	223	0	Health and fitness products represented 60% of the inventory.
2009	1015	212	107	Added archiving function to the CPI.
2010	1015	0	0	No data collected.
2011	1015	0	0	No data collected.
2012	1438	426	0	Beginning of CPI 2.0 project, focus on adding new products.
2013	1628	190	288	Launch of crowdsourcing component. Extensive effort put into adding and archiving products.
2014	1814^a^	238^a^	223^a^	Extensive effort put into adding and archiving products.

^a^The CPI now has crowdsourcing capabilities, so these numbers are a snapshot in time and will not represent the CPI at the time of reading.

The products listed on the CPI 2.0 satisfy three criteria: (1) they can be readily purchased by consumers; (2) they are claimed to contain nanomaterials by the manufacturer or another source; and (3) their claim to contain nanomaterials appears reasonable to CPI curatorial staff.

Although the steady growth of the inventory indicates that the popularity of products claimed to incorporate nanotechnology is continually increasing, not all products have persisted in the consumer market. In the past seven years, 34% of the entries in the inventory have been archived because the product is not currently available in the market or their claim to contain nanotechnology can no longer be verified. One example of a claim that can no longer be verified is a product that is still available for purchase on a manufacturer’s website but no longer references, explicitly, the incorporation of nanotechnology into that product. Even after archiving, a product can return to the main inventory listing if a third party makes the claim that the product indeed contains nanomaterials or if the manufacturer restates their nanomaterial claim.

**Figure 1:**
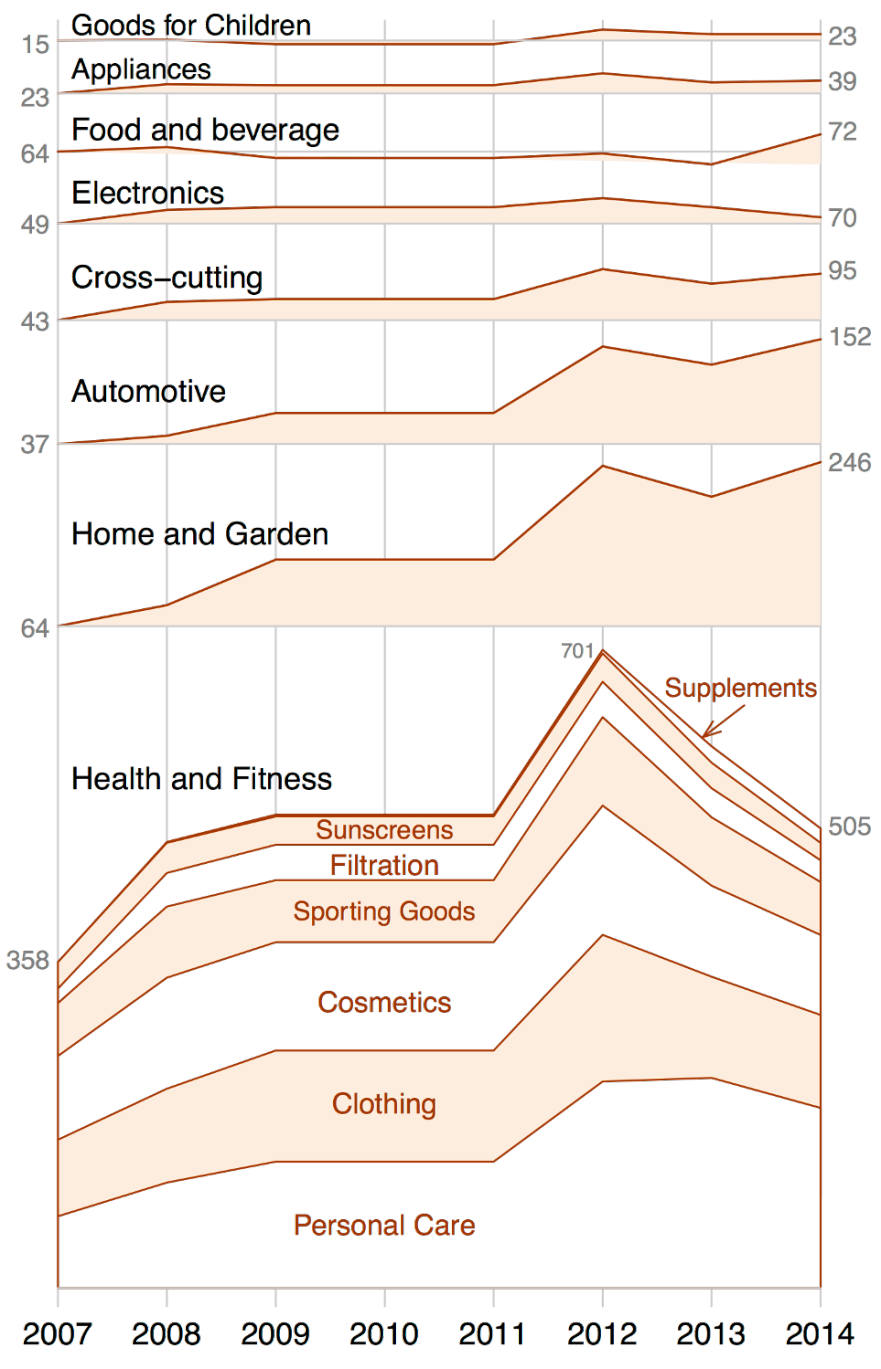
Number of available products over time (since 2007) in each major category and in the Health and Fitness subcategories.

In the CPI, entries are grouped under eight generally accepted consumer goods categories that are loosely based on publicly available consumer product classification systems (Figure 1) [19]. The Health and Fitness category includes the largest listing of products in the CPI, comprising 42% of listed products (excluding archived products). Within the Health and Fitness category, Personal Care products (e.g., toothbrushes, lotions, and hairstyling tools and products) comprise the largest subcategory (39% of products). Starting in 2012, a large continual effort has been put into periodically checking products for their current availability and current claim to contain nanotechnology. This effort resulted in archiving 316 products in the Health and Fitness category – mainly in the Personal Care and Clothing subcategories – with 86 and 78 products archived between 2012 and 2014, respectively.

### New nanomaterial descriptors

Eight new product descriptors were introduced to facilitate the use of this database by a variety of stakeholders (namely industry and the scientific and regulatory communities):

1. main nanomaterial composition or type,
2. nanomaterial shape and size,
3. nanomaterial coating or stabilizing agent,
4. nanomaterial location within the product,
5. nanomaterial function in the product,
6. potential exposure pathways,
7. “how much we know”,
8. “researchers say”.

The experimental section of this paper describes all new product descriptors. The results of the five new quantitative descriptors are presented and discussed below. Since the “nanomaterial shape and size”, “coating and stabilizing agent”, and the “researchers say” categories are text-entry data fields, thus qualitative information at this point, we have not included their analysis in this paper.

### Nanomaterial composition

**Figure 2:**
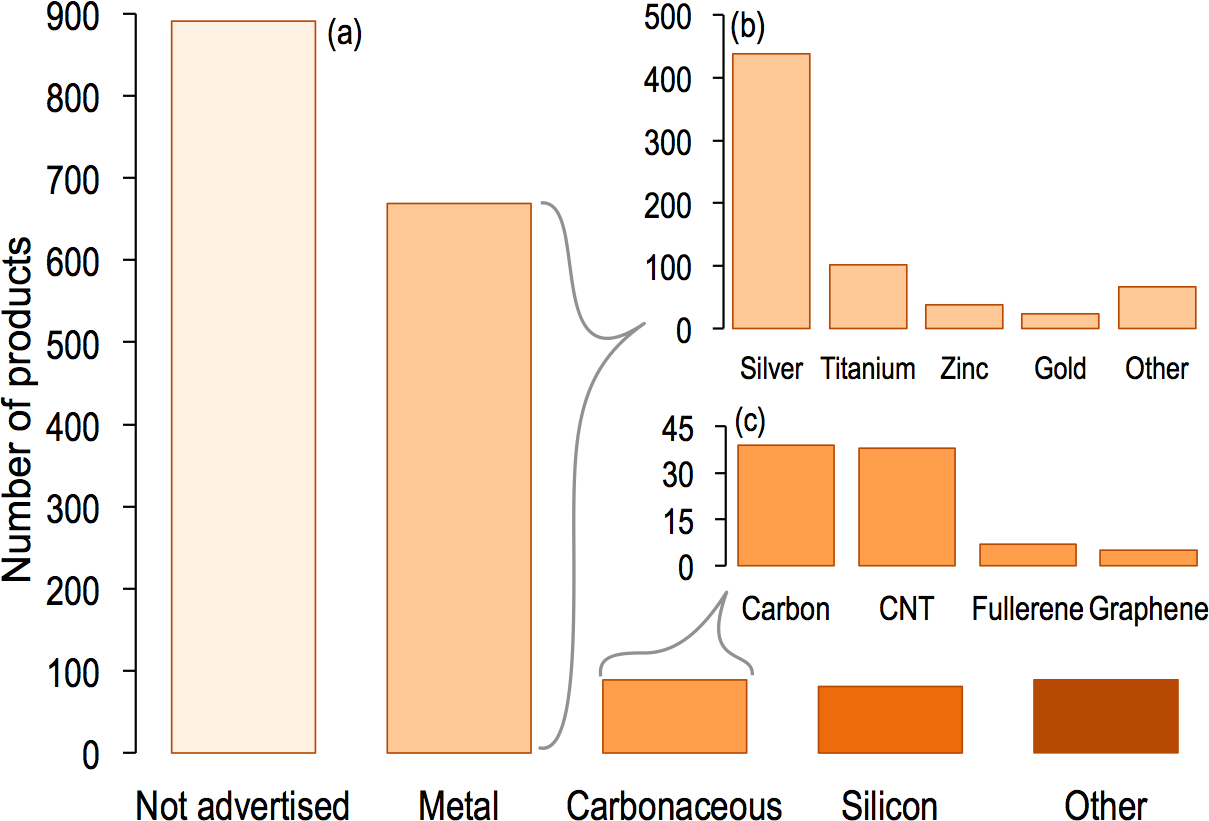
(a) Claimed composition of nanomaterials listed in the CPI, grouped into five major categories: not advertised, metal (including metals and metal oxides), carbonaceous nanomaterials (carbon black, carbon nanotubes, fullerenes, graphene), silicon-based nanomaterials (silicon and silica), and other (organics, polymers, ceramics, etc.). (b) Claimed elemental composition of nanomaterials listed in the metals category: silver, titanium, zinc, gold, and other metals (magnesium, aluminum oxide, copper, platinum, iron and iron oxides, etc.). (c) Claimed carbonaceous nanomaterials (CNT = carbon nanotubes).

Of the 1814 products listed in the CPI, 47% (846 products) advertise the composition of at least one nanomaterial component and 62 of those products list more than one nanomaterial component (e.g., a product comprised of both silver and titanium dioxide nanomaterials). There are 39 different types of nanomaterial components listed in the inventory (listed in Supporting Information File 1, Table S2), which have been grouped into five major categories in Figure 2 and Figure 4, to improve their legibility: metal, carbonaceous, silicon, not advertised, and other. Nominally, metals and metal oxides comprise the largest nanomaterial composition group advertised in the inventory, listed in 37% of products.

Titanium dioxide (TiO_2_), silicon dioxide, and zinc oxide are the most produced nanomaterials worldwide (on a mass basis) and the global annual production of silver nanoparticles represents only 2% of that of TiO_2_ [20–21]. However, silver nanoparticles are the most popular advertised nanomaterial in the CPI, present in 438 products (24%). The CPI reports the numbers of different consumer products and product lines available in the market, so there is no implication on mass, volume, or concentration of nanomaterials incorporated into products or the production volume of each product.

Of carbonaceous nanomaterials (89 products), the majority of products listed contains carbon nanoparticles (sometimes described as carbon black, 39 products) and single- or multi-walled carbon nanotubes (CNT, 38 products). Unfortunately, 891 (49%) of the products included in the CPI do not present the composition or a detailed description of the nanomaterial used (Figure 2).

The percentages of nanomaterial compositions in the CPI 2.0 are somewhat in agreement with those of the Danish Nanodatabase. The Nanodatabase also lists a high fraction of products with unknown nanomaterial composition (944 products or 66%) and, among known compositions, silver is also the most frequently advertised nanomaterial component, with 207 products or 14.5% [11]. Silver nanoparticles are popular consumer product additives due to their well-documented antimicrobial properties [22].

**Figure 3:**
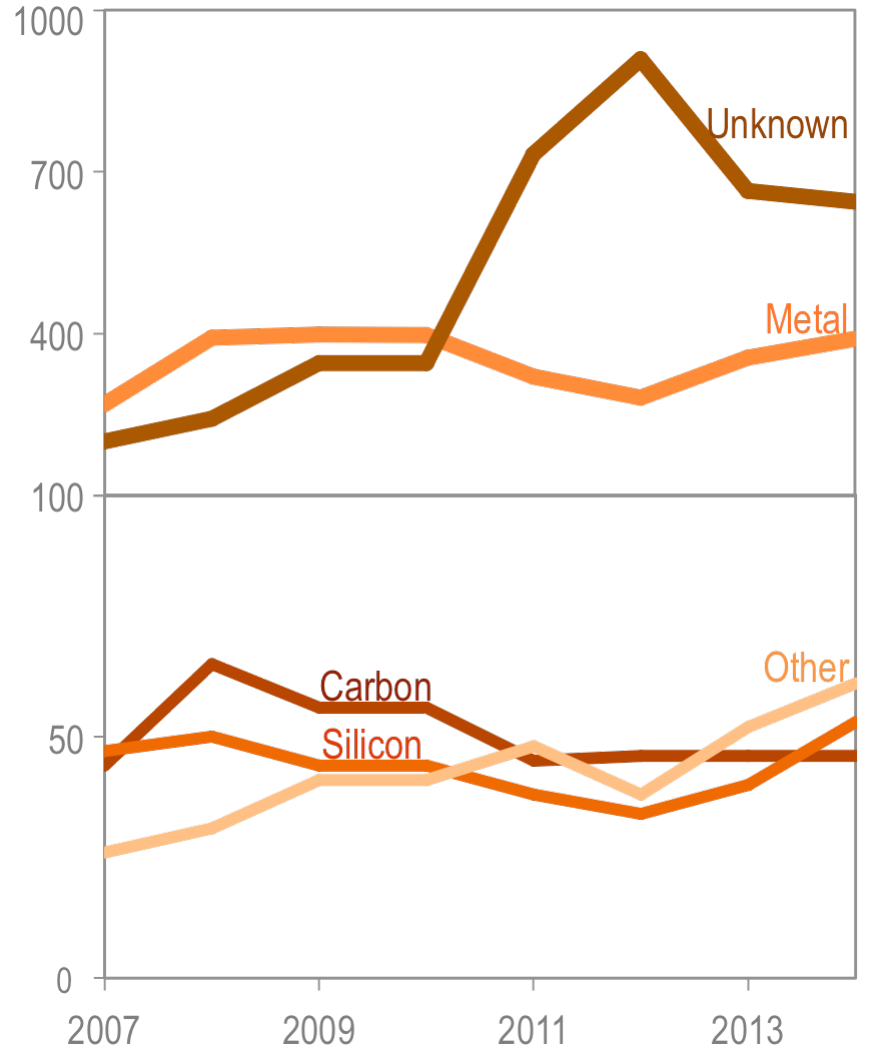
Major nanomaterial composition groups over time. Carbon = carbonaceous nanomaterials (carbon black, carbon nanotubes, fullerenes, graphene). Other = organics, ceramics, polymers, clays, nanocellulose, liposomes, nano micelles, carnauba wax, etc. Note the difference in scale between the top and bottom panels in this plot.

Figure 3 shows how the availability of these major nanomaterial composition groups changed over time. Since the start of the CPI 2.0 project (2012), products with unknown (not advertised) nanomaterial compositions have decreased by 12%, which is partially due to these products being archived and of their composition being identified and added to the inventory. Products advertising to contain metal and metal oxide nanomaterials, silicon-based nanomaterials (mostly SiO_2_ nanoparticles), and a variety of other nanomaterial components (organics, ceramics, polymers, clays, nanocellulose, liposomes, nano micelles, carnauba wax, etc.) have been growing in popularity. During the same period, carbonaceous nanomaterials have remained stable at around 50 products available in the market.

**Figure 4:**
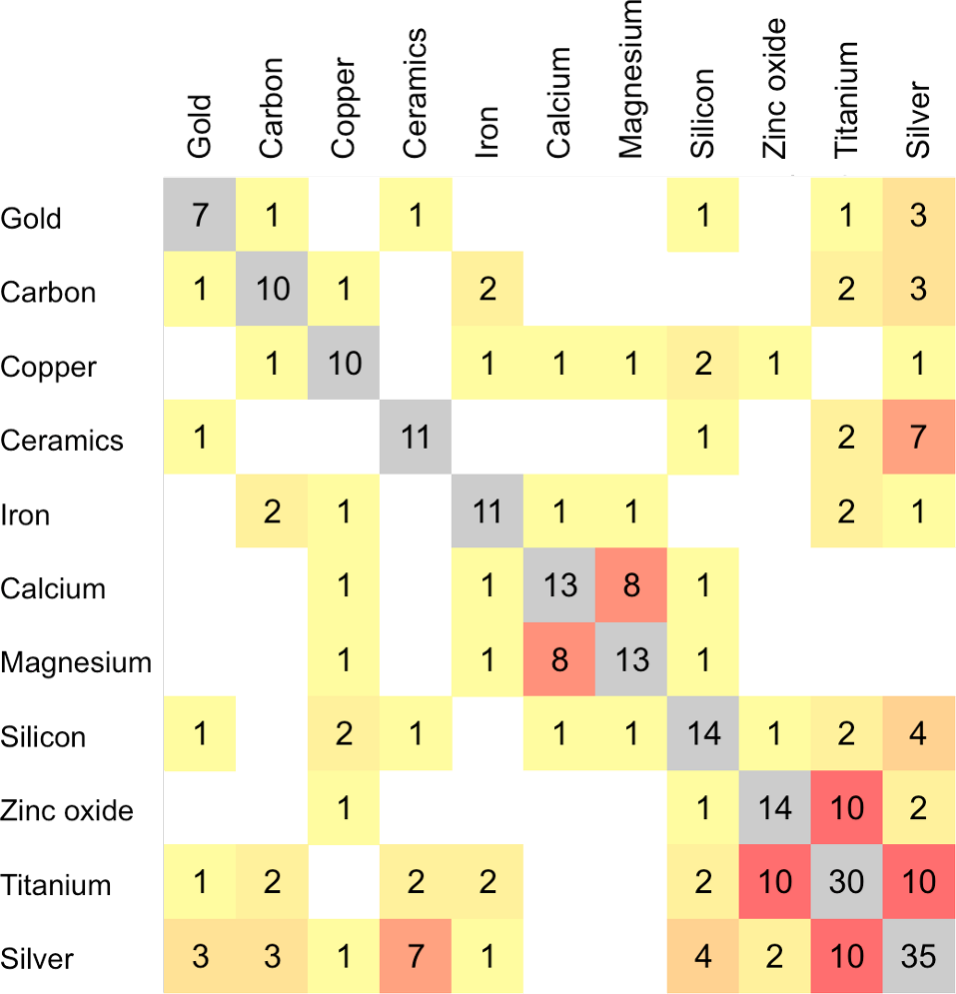
Major nanomaterial composition pairs in consumer products. Carbonaceous nanomaterials (carbon black, carbon nanotubes, fullerene, and graphene) were combined into the same category (carbon). Grey boxes in the diagonal represent the total times each nanomaterial composition has been listed with other compositions in the same product.

Of the 846 products listed in the CPI for which we were able to determine a nanomaterial composition, 61 products (7%) advertise to contain more than one main nanomaterial component. Figure 4 presents 11 nanomaterial components that were most frequently listed with others in the same product.

Silver and titanium dioxide are the nanomaterial components most likely to be combined with other nanomaterials in consumer products, with 35 and 30 product combinations, respectively. Silver and titanium dioxide were paired with each other in 10 products (cosmetics and electronics); titanium dioxide and zinc oxide were paired in 10 products (sunscreens, cosmetics, and paints). The European Commission’s Cosmetics Regulation has permitted the use of nanoscale titanium dioxide in sunscreens, but not zinc oxide [17].

Calcium and magnesium were listed together in dietary supplements. Nano-ceramics and silver are used in combination in water filtration products, cosmetics, and a humidifier. These results demonstrate the use of nanohybrids [23] in consumer products and indicate that the use of nanotechnology-based consumer products in the home may, in some cases, lead to multiple exposures from a combination of nanomaterial compositions. These results suggest the need to examine nanomaterial toxicity effects that could be synergistic, additive, or even antagonistic.

### Nanomaterial location

**Figure 5:**
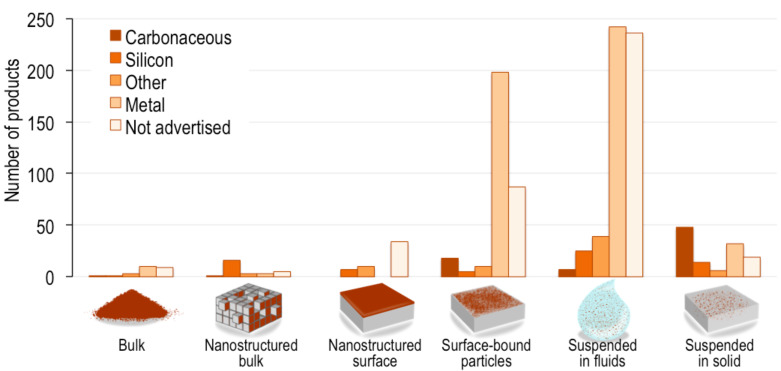
Locations of nanomaterials in consumer products for which a nanomaterial composition has been identified.

About 29% of consumer products in the CPI (528 products) contain nanomaterials suspended in a variety of fluids (e.g., water, skin lotion, oil, car lubricant). The second largest group in this category – with 307 products – comprises solid products with surface-bound nanoparticles (e.g., hair curling and flat irons, textiles). Figure 5 shows the location of nanomaterials for which a composition has been identified [24].

The majority (64%) of carbonaceous nanomaterials are embedded in solid products, whereas products of all other compositions are more commonly suspended in liquid. Of the few bulk nanomaterials that are available for purchase by consumers, the largest group (42%) consists of metal and metal oxide nanomaterials. Metals and metal oxides were also the largest composition for surface-bound particles and those suspended in liquid products. The majority (67%) of products with nanostructured surfaces consist of nanomaterials of undetermined composition. An example of such product is a liquid or spray products that forms a nanofilm upon application over a surface. Of nanostructured bulk materials, the majority (57%) are silicon-based nanomaterials (e.g., computer processor parts). It is interesting to note that we expect nano-electronics to exist now in massive numbers of consumer products, such as mobile devices, where field effect transistors, the heart of chip technology, have components (sources, gates, collectors, channels) that are now in the nanoscale [25] and would fit into the nanostructured bulk category. However, because most of these products do not advertise their use of nanomaterials, we believe that they are grossly underrepresented in the CPI.

### Nanomaterial function

**Figure 6:**
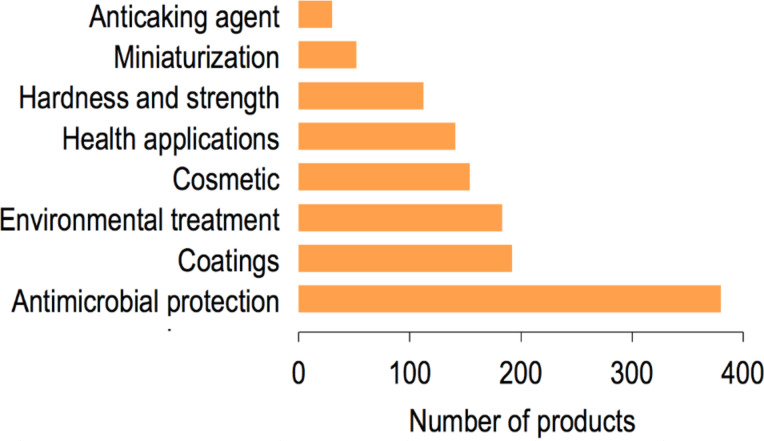
Expected benefits of incorporating nanomaterial additives into consumer products.

Of the 1814 inventory entries, 1244 were grouped according to the expected benefits of adding such nanomaterials to the product (Figure 6). A significant portion of products in the CPI (31% of products analyzed) utilize nanomaterials – mostly silver nanoparticles, but also titanium dioxide and others – to confer antimicrobial protection. Nanomaterials such as titanium dioxide and silicon dioxide are used to provide protective coatings (15%) and for environmental treatment (to protect products against environmental damage or to treat air and water in the home, 15%). Cosmetic products (12%) are advertised to contain a variety of nanomaterials such as silver nanoparticles, titanium dioxide, nano-organics, gold, and others. A wide variety of nanomaterial compositions (silver, nano-organics, calcium, gold, silicon dioxide, magnesium, ceramics, etc.) were also advertised to be used for health applications, such as dietary supplements (11%).

### Potential exposure pathways

**Figure 7:**
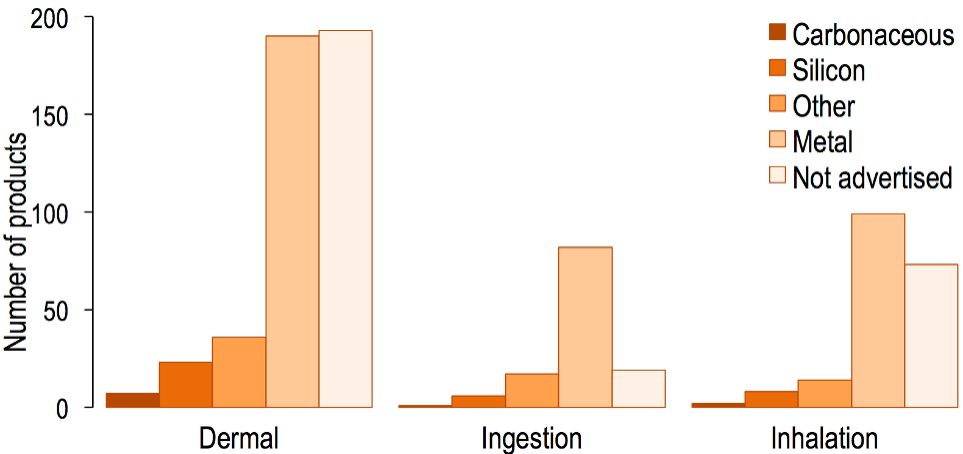
Potential exposure pathways from the expected normal use of consumer products, grouped by major nanomaterial composition categories.

Since critical information such as nanomaterial size and concentration are not known for most products listed on the CPI, the actual health risks of these products remain largely unknown. Nevertheless, the CPI may be useful for inferring potential exposure pathways from the expected normal use of listed products. To investigate this utility, we analyzed a subset of 770 products from the CPI to determine their most likely route(s) of exposure (Figure 7).

We identified the skin as the primary route of exposure for nanomaterials from the use of consumer products (58% of products evaluated). This is because many entries in the CPI consist of (1) solid products that contain nanomaterials on their surfaces and are meant to be touched or (2) liquid products containing nanomaterial suspensions which are meant to be applied on the skin or hair. Of the products evaluated, 25% present nanomaterials that can possibly be inhaled during normal use (e.g., sprays and hair driers) and 16% contain nanomaterials that may be ingested (e.g., supplements and throat sprays). Hansen et al. developed a framework for exposure assessment in consumer products. In this framework, products that contain nanomaterials suspended in liquid and products that may emit airborne nanoparticles during use are expected to cause exposure [26].

Since metals and metal oxides are the most common nanomaterial composition in the CPI, they are also the most likely materials to which consumers will be exposed during the normal use of product via dermal, ingestion, and inhalation routes. Products containing nanomaterials of unknown composition are most likely to lead to exposure via the dermal route.

Berube et al. [7] offered a critique of the original CPI in 2010, which focused primarily on the lack of data pertinent to the dosages of nanomaterials to which consumers might be exposed through CPI-listed products. This is a valid criticism given that information used to populate the CPI is based primarily on marketing claims made by manufacturers. However, the most recent modifications of the CPI offer a potential remedy for data gaps through the contributions of third-party research teams. These modifications are especially timely as there is a growing number of published studies assessing consumer exposure to nanomaterials released during the use of nanotechnology-enhanced consumer products [27], such as cosmetic powders [28], sprays [29–30], general household products [31], and products for children [32–33]. One challenge is that there are no standardized methods for assessing consumer risks from using nanotechnology-enabled consumer products or a set of agreed-upon metrics for characterizing nanomaterials to determine environmentally relevant concentrations [34]. The development of such standards is seen as a top strategy for safe and sustainable nanotechnology development in the next decade [35]. The Consumer Product Safety Commission recently requested $7 million to establish the Center for Consumer Product Applications and Safety Implications of Nanotechnology to help develop methods to identify nanomaterials in consumer products and to understand human exposure to those materials [36].

### How much we know

**Figure 8:**
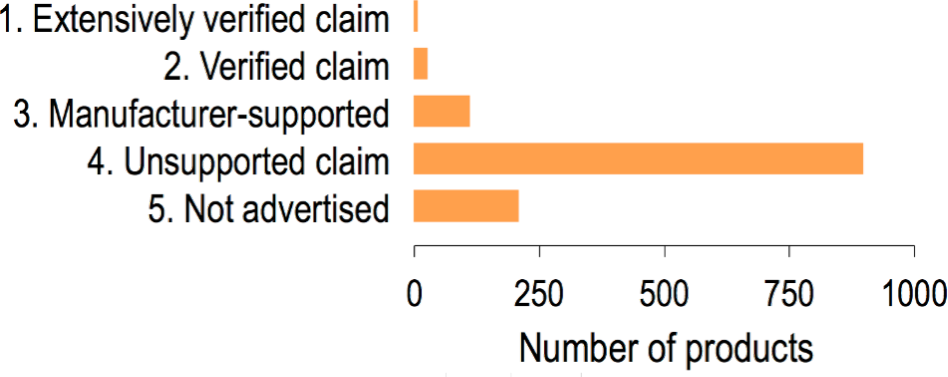
Distribution of products into the “How much we know” categories.

Through the “How much we know” descriptor, inventory entries are rated according to the reliability of the manufacturer’s claim that products contain nanomaterials. We evaluated 1259 products present in the inventory for the “How much we know” descriptor and the majority (71%) of products are not accompanied by information sufficient to support claims that nanomaterials are indeed used in the products, such as a manufacturer datasheet containing technical information about nanomaterial components (e.g., median size, size distribution, morphology, concentration). Only nine products have been classified in Category 1, “Extensively verified claim” due to the availability of scientific papers or patents describing the nanomaterials used in these products (Figure 8). The experimental section, below, presents a full description of these categories.

Hansen [37] performed interviews with 26 nanotechnology stakeholders who agreed on an incremental approach to nanomaterial regulation in consumer products, including classification and labeling. The European Commission’s Classification, Labeling, and Packaging (CLP) regulation covers nanomaterials that are classified by the Commission as hazardous chemical substances [15]. Becker [38] reported that there are diverging opinions in the nanotechnology industry with regards to labeling, ranging from ‘‘If it’s a nano-scale material, people should know, hands down” to not supporting labeling because “it wouldn’t accurately inform consumers of anything and would be bad for business because it would scare consumers.”

Appropriate nanomaterial labeling containing sufficient technical information (i.e., at a minimum, nanomaterial composition, concentration, and median size) would better inform consumers and highly benefit researchers interested in understanding consumers’ exposure and nanomaterial fate and transport in the environment.

### Crowdsourcing

Since October 29, 2013, when the modified inventory (CPI 2.0) was released, 557 new user accounts have been requested. Of these, only approximately 10 users who were not directly or indirectly involved in the research team performing the CPI upgrade and maintenance suggested updates or edits to CPI entries. These edits have all been suggested by users from industry and academia.

Future work is needed to better educate users on their role as curators of CPI 2.0 and the importance of the data they contribute. Providing the supporting technical data required to verify the nature and quantity of nanomaterial components in CPI-listed products is a massive undertaking, and no single laboratory can accomplish it on its own or within a short amount of time. A long-term solution is to promote the importance of crowd-sourcing data collection and implementing standard data collection and reporting best practices that can help reliably populate the CPI with much needed supporting data. The new crowd-sourcing capability can also be used to provide high school-, undergraduate- and graduate-level educators with meaningful assignments that can help teach students about the prevalence of nanotechnology in everyday products and will contribute to the continued growth of this resource.

### Nanotechnology expert survey

The survey was submitted to 147 people who have published research papers or reports in the applications of nanotechnology in consumer products and its potential impacts, participated in recent conferences in the field, or were notably involved in the field of nanotechnology and the consumer products industry. The survey had a 46% response rate (68 respondents), which is in the expected range for this type of survey [39]. The majority of respondents (59%) had six to ten years of experience working with nanotechnology and 38% of respondents had more than ten years of experience. Half (51%) of respondents work in academic institutions and 25% work in governmental agencies. Most respondents (88%) have previously used the CPI in their work, and all respondents believe they will or may use it again in the future.

**Figure 9:**
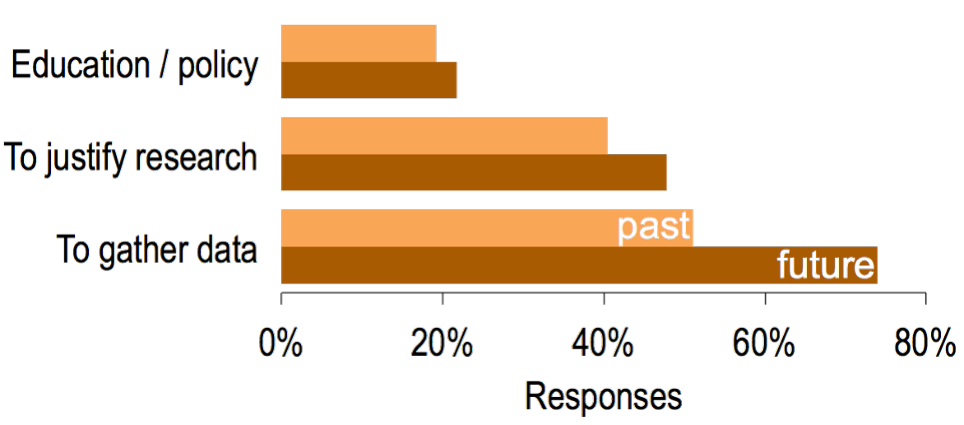
Nanotechnology survey answers on how respondents have used the CPI in the past and how they might use it in the future.

Results convey a general belief or hope that the CPI will become more useful after the modifications reported in this publication. When asked the following open-ended questions: “How did you use the CPI in your work?” and “To what end do you think you might use the CPI in the future?”, answers could be easily grouped into three main categories: (1) for raising awareness, teaching, or for urging the need for regulation, (2) to justify the need for research in research proposals or papers, and (3) to use the inventory data for research (Figure 9).

Half the respondents (51%) have used the CPI in the past to gather data for research (e.g., searching for consumer products of a certain nanomaterial composition to understand their potential applications or consumer exposure) while 74% believe they will use the CPI for that purpose in the future. The majority (79%) of survey respondents believed the modified CPI would present more products than its previous version, which indicates their belief in the growing prevalence of nanotechnology in consumer products.

Survey respondents suggested a number of new categories of information for the CPI 2.0, including nanomaterial type or composition, location of nanomaterial within the product, nanomaterial size, relevant scientific publications that describe the products in the inventory, a summary of known toxicity of the advertised nanomaterial, supply chain information, volume produced, and life cycle assessment information.

Most of these suggestions were included in the CPI 2.0 as the new categories described in this work. Others, such as known nanomaterials toxicity were not pursued since toxicity can vary greatly depending on particle size, coating, and exposure route (e.g., inhalation versus ingestion).

Piccinno et al. and Keller et al. provide global estimates for production and major applications of nanomaterials [20–21]. We recommend that future work associated with this inventory or others include information on the production volumes for each product, since this information is presently unavailable.

Additional results from this survey are available in Supporting Information File 1.

## Conclusion

The modified version of the Wilson Center’s nanotechnology consumer products inventory (CPI 2.0) was released in October 2013. We improved the searchability and utility of the inventory by including new descriptors for both the consumer products and the nanomaterial components of those products (e.g., size, concentration, and potential exposure routes). The updated CPI 2.0 now links listed products to published scientific information, where available, and includes a metric to assess the reliability of the data associated with each entry. Finally, the CPI 2.0 has enabled crowdsourcing capabilities, which allow registered users to upload new findings such as basic product composition information, human and environmental exposure data, and complete life cycle assessments. There are inherent limitations to this type of database, but recent improvements address the majority of issues raised in published literature and in a survey of nanotechnology experts.

Improvements to the CPI were motivated, in part, by the recognition that it represents and will continue to represent an important information resource for a broad range of stakeholders, especially consumers and the academic and regulatory communities. The CPI is a useful interactive database for educating consumers and legislators on the real-world applications of nanotechnology. Michaelson stated that the CPI transformed “the face of nanotechnology away from innovations in the realm of science fiction to the iconic images of everyday consumer products” [2]. The academic community can continue to make use of this inventory to help prioritize, for example, which types of products or nanomaterial components to evaluate in human exposure or toxicity studies, life cycle assessments, and nanomaterial release studies.

The CPI is useful for policy makers interested in regulating nanotechnology in consumer products by understanding their increasing numbers in the market, the main nanomaterial components that are chosen by manufacturers, and the likelihood for exposure. Beaudrie et al. [40] urge that there should be regulatory reforms to improve oversight of nanomaterials throughout their life cycle.

Finally, the current lack of global standardized methods and metrics for nanomaterial characterization and labeling in consumer products is an issue that, if addressed, can lead to greater understanding between the key stakeholders in nanotechnology, especially researchers, regulators, and industry. Further, as we recognize the growing importance of tools like the CPI for the needs of diverse stakeholder groups, steps should be taken to help ensure that those tools are fully developed and refined to meet those needs.

## Experimental

### Nanotechnology expert survey

To determine potentially useful improvements for the CPI, we developed a web-based survey to gather the informed opinions of nanotechnology experts – mostly in US-based academic institutions, governmental agencies, and research centers. Their answers guided the CPI modifications and provided an idea of the expectations related to the inventory. The survey questions are presented in the Supporting Information File 1.

### New descriptors

To improve the utility and searchability of this database, seven product descriptors were created. Entries in the inventory were revised to go beyond a categorization of the consumer products and instead, to include more information on the nanomaterials themselves. We searched for this information mainly on the internet – on manufacturer’s websites, retailer’s websites, news sites and blogs, patents – and, when available, product labels.

### Nanomaterial composition

The main composition of the nanomaterials used. This information, when available, was added to the database in the form of a check-box list, in which more than one nanomaterial composition can be selected for each consumer product.

### Nanomaterial shape and size

Because there are many different ways in which manufacturers can measure and describe the shape and size of nanomaterials in consumer products (i.e., units of nanometers or micrometers, thickness of nanofilms, diameter or length of fibers or tubes, diameter or radius of nanoparticles, maximum, median, average, or minimum size), this descriptor was added as a text entry field in the database, which allows for any form of data entry but makes data analysis cumbersome.

### Coatings

We created another text entry field in the CPI to include any available information on the coatings or stabilizing agent used along the nanomaterials in each product.

### Nanomaterial location

To assist CPI users in understanding the potential for nanomaterial release and exposure scenarios from the use of these consumer products, we created a qualitative descriptor for the location of nanomaterials within each product. We adapted the categorization framework for nanomaterials from Hansen et al. [24] to determine the following nanomaterial locations within products:

- Bulk: Nanomaterials sold in powder form or in liquid suspensions
- Nanostructured bulk: Products or parts that contain nanostructured features in bulk (e.g., nanoscale computer processors)
- Nanostructured surface: Products or parts that contain nanostructured features on their surface (e.g., nanofilm-coated products)
- Surface-bound particles: Nanoparticles added to the surface of a solid product or part (e.g., a computer keyboard coated with silver nanoparticles for antimicrobial protection)
- Suspended in liquid: Nanomaterials suspended in a liquid product (e.g., disinfecting sprays, liquid supplements)
- Suspended in solid: Nanomaterials suspended in a solid matrix, usually plastic or metal (e.g., composites of carbon nanotubes in a plastic matrix to confer strength).

### Nanomaterial function

We created a metric to describe the reason why nanotechnology was added to each consumer product or the function it performs within each product. We investigated a subset of 1244 products in the CPI for each product’s intended use, the manufacturer claims, and, most importantly, the type or composition of nanomaterials used to infer potential nanomaterial functions (e.g., antimicrobial protection, hardness and strength, pigment).

### Potential exposure pathways

Using methodology similar to that applied for the “nanomaterial functions” category, we investigated the CPI entries for possible exposure scenarios resulting from the expected normal use of each consumer product. Entries were only populated when a potential exposure risk was identified.

### How much we know

In an effort to verify the data associated with each product listed on the CPI, we created a metric called “How much we know”. Products were divided into five categories based on the information available to substantiate manufacturer claims that a particular product contains nanomaterial components (Table 2). Category 4, “Unsupported claim”, is the default category for products added to the CPI based soley on a manufacturer’s marketing claims. A product can rise in ranking according to the amount of information that is available to corroborate the manufacturer’s claim that the product contains nanomaterials. If the manufacturer provides supporting information (e.g., a datasheet containing electron micrographs showing the nanomaterials or a particle size distribution), the product is placed in Category 3, “Manufacturer-supported claim”. If a third-party further supports the information provided by the manufacturer, such as through a publication or technical report, then the product can be placed into Category 2, “Verified claim”. If a product is backed by multiple science-based sources (e.g., a peer-reviewed scientific paper or patent documentation), it is then placed in Category 1, “Extensively verified claim”. Category 5, “Not advertised by the manufacturer”, is a special class for products that have been shown to contain nanomaterials but the manufacturer does not advertise this fact anywhere in product labeling or other informational materials. Category 5 has been added in recognition of the fact that not all nano-enabled products are marketed by manufacturers as such.

**Table 2:** “How much we know” categorization, based on the information available to substantiate manufacturer claims that a particular product contains nanomaterial components.

Category	Manufacturer claims to use nanotechnology	Manufacturer provides supporting information	Third-party information is available	Compelling information from multiple sources is available

1. Extensively verified claim	yes	yes	yes	yes
2. Verified claim	yes	yes	yes	
3. Manufacturer-supported claim	yes	yes		
4. Unsupported claim	yes			
5. Not advertised by manufacturer			yes	

### Researchers say

In order to add available scientific information to the inventory, we created a text-entry database field named “Researchers say”, which makes it possible to include an extract from a research paper (such as the abstract), author citation, and a link to the paper.

### Crowdsourcing

We added a new crowdsourcing capability to the CPI website so that consumers, manufacturers, and the greater scientific community can contribute new information on nanomaterial composition of CPI products to the inventory. New contributors must request an account by completing a form with their contact information, and they must provide a reason why they would like to gain access to this crowdsourcing tool. Accounts are manually reviewed. Access is granted to all requesters who complete the form and have a legitimate purpose for contributing information. Once an account is created, users may sign in and suggest edits to any product (including the archiving of products no longer available or no longer advertising to contain nanomaterials) or suggest new products to the inventory. As a quality control measure, suggestions and new product forms contributed by registered users must be approved by a CPI curator before updates or revisions are posted to the inventory.

## Supporting Information

File 1 A compilation of company and product numbers listed by country of origin. A list of all nanomaterial components included in the inventory. Nanotechnology expert survey questions. Additional nanotechnology expert survey results.
