# Supplementary material for: Nanotechnology in the real world: Redeveloping the nanomaterial consumer products inventory
Source: Beilstein J Nanotechnol. 2015 Aug 21;6:1769–80. doi: 10.3762/bjnano.6.181 (PMC4578396; doi:10.3762/bjnano.6.181)
Supplement: File 1 — A compilation of company and product numbers listed by country of origin. A list of all nanomaterial components included in the inventory. Nanotechnology expert survey questions. Additional nanotechnology expert survey results. [file Beilstein_J_Nanotechnol-06-1769-s001.pdf]

Supporting Information

for

Nanotechnology in the real world: Redeveloping the nanomaterial consumer products inventory

Marina E. Vance[*1], Todd Kuiken[2], Eric P. Vejerano[3], Sean P. McGinnis[4], Michael F. Hochella, Jr.[5], David Rejeski[2] and Matthew S. Hull[1]

Address: [1]Institute for Critical Technology and Applied Science, Virginia Tech, 410 Kelly Hall (0194), 235 Stanger St., Blacksburg, VA 24061, United States, [2]Woodrow Wilson International Center for Scholars, One Woodrow Wilson Plaza - 1300 Pennsylvania Ave., NW, Washington, DC 20004, United States, [3]Department of Civil & Environmental Engineering, Virginia Tech, 418 Durham Hall (0246), Blacksburg, VA 24061, United States, [4]Department of Materials Science and Engineering, Virginia Tech, Holden Hall (0237), Blacksburg, VA 24061, United States and [5]Department of Geosciences, Virginia Tech, 4044 Derring Hall (0420), Blacksburg, VA 24061, United States

Email: Marina E. Vance - marinaeq@vt.edu

* Corresponding author

Contents:

1. Number of products per country of origin
2. Nanomaterial compositions listed in the inventory
3. Nanotechnology expert survey questions
4. Additional nanotechnology expert survey results

1. Number of products per country of origin

Table S1: Number of companies and products per country of origin.

	Country	Companies	Products		Country	Companies	Products
1	USA	290	773	18	Finland	3	3
2	Korea	64	132	19	Malaysia	3	4
3	Germany	63	328	20	Singapore	3	24
4	UK	35	104	21	Czech Republic	2	16
5	Japan	32	56	22	Netherlands	2	5
6	China	24	57	23	New Zealand	2	5
7	Denmark	12	47	24	Sweden	2	2
8	Australia	10	21	25	Argentina	1	1
9	France	10	34	26	Brazil	1	1
10	Taiwan	10	28	27	Iceland	1	1
11	Canada	9	17	28	India	1	2
12	Italy	7	14	29	Ireland	1	1
13	Switzerland	7	44	30	Lithuania	1	1
14	Israel	6	10	31	Mexico	1	1
15	Austria	4	11	32	Philippines	1	1
16	Poland	4	17		Unknown	6	49
17	Thailand	4	4		**Total**	**622**	**1814**

2. Nanomaterial compositions

Table S1: Nanomaterial components listed in the inventory.

Aluminum oxide	Gold	Nickel
Boron	Graphene	Organics
Calcium	Graphite	Palladium
Carbon	Iodine	Platinum
Carbon nanotube	Iridium	Polymer
Carnauba wax	Iron	Retinol
Ceramics	Lead	Silicon
Cerium oxide	Liposome	Silver
Chromium	Lithium	Titanium
Clay	Magnesium	Tungsten disulfide
Cobalt	Manganese	Zeolite
Copper	Nano micelles	Zinc oxide
Fullerene	Nanocellulose	Zirconia

3. Nanotechnology expert survey questions

Part I

Tell us about your expectations for the CPI 2.0.

1. How many products do you expect CPI 2.0 will contain? *(about 1,300 products are currently listed, some of which may no longer exist)*

- ☐ Fewer products (< 1,200)
- ☐ About the same amount of products (~ 1,200 - 1,500)
- ☐ More products (~ 1,500 - 2,000)
- ☐ A lot more products (> 2,000)

2. Have you used the CPI in your work?

- ☐ Yes
- ☐ No

3. How did you use the CPI in your work?

– text answer –

4. Do you think you will use the new CPI in your work?

- ☐ Yes
- ☐ Maybe
- ☐ No

5. To what end do you think you might use the CPI in the future?

– text answer –

Part II

6. Please suggest new categories of information (describing products or nanomaterials within products) to be added to the CPI.

Current categories present in the inventory are:

- Country or manufacture
- Company name
- Product category (e.g., automotive, food and beverage, goods for children)
- Date added to the inventory

Here are a few examples of new categories to be added:

- Type of nanomaterial used in the product (e.g., silver nanoparticles, carbon nanotubes)
- Location of nanomaterial within the product (e.g., coating, suspended in liquid).

– text answer –

Part III

Demographic questions.

7. Your Name *(personal identifying information will not be published in any form)*

– text answer –

8. Your organization *(identifying information will not be published)*

– text answer –

9. Your years of experience working with nanotechnology:

- ☐ 0 - 2 years
- ☐ 3 - 5 years
- ☐ 6 - 10 years
- ☐ 11 - 19 years
- ☐ 20+ years

10. How can you describe your organization? *(please check all that apply)*

- ☐ Science Community (university)
- ☐ Science Community (non-university)
- ☐ Nanotechnology Industry
- ☐ Non-nanotechnology Industry
- ☐ Government Agency
- ☐ Non-Governmental Agency
- ☐ Consumer Advocacy Group
- ☐ Other

11. Please enter any additional comments here:

– *text answer* –

4. Selected nanotechnology expert survey results

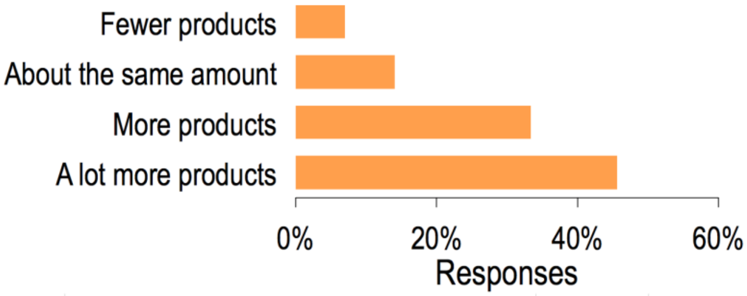

Figure S1: Question 1, “How many products do you expect CPI 2.0 will contain?”.

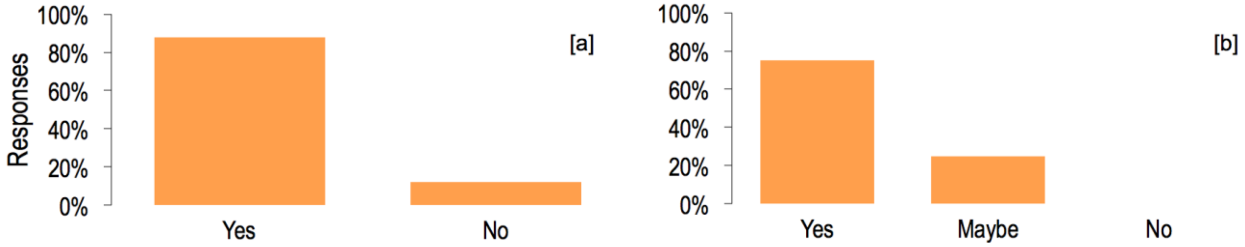

Figure S2: (a) Question 2, “Have you used the CPI in your work?” (b) Question 4, “Do you think you will use the new CPI in your work?”.

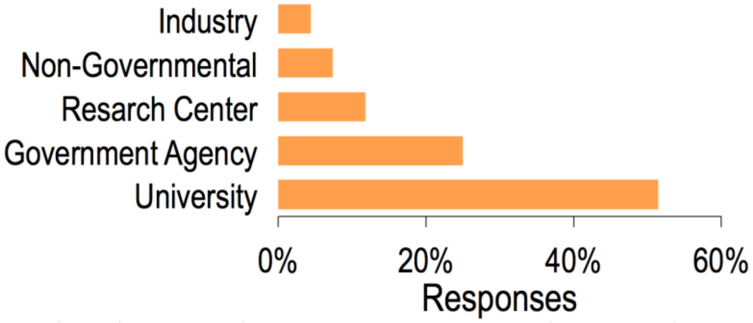

Figure S3: Question 10, “How can you describe your organization?”.
